# Imaging Characteristics and Diagnostic Implications of Breast Amyloidoma

**DOI:** 10.1155/crra/8480457

**Published:** 2025-04-16

**Authors:** Michael F. Chan, Tamarya L. Hoyt

**Affiliations:** Department of Radiology and Radiological Sciences, Vanderbilt University Medical Center, Nashville, Tennessee, USA

## Abstract

Breast amyloidoma is an extremely rare and potentially underdiagnosed entity with underreported diagnostic implications. Breast amyloidomas typically present as painless masses with varying imaging characteristics ranging from nonspecific asymmetries to mammary carcinoma mimics. Calcifications are visualized in many described cases in the literature with a characteristic histopathologic appearance following Congo red staining under polarized light. While breast amyloidomas are benign lesions, there are proposed associations with systemic autoimmune and hematolymphoid disorders in the literature. This suggests the need for standardized management parameters following diagnosis, as the treatment of localized amyloidosis differs greatly from that of its systemic counterpart. To date, no consensus guidelines for follow-up or management of breast amyloidomas exist, owing to its rarity and dearth of cases in the literature. Due to the necessity of employing specific staining to establish the diagnosis, underdiagnosis and occasionally misdiagnosis may contribute to its apparent rarity. In this radiologic–pathologic correlation, a unique case of breast amyloidoma is presented to highlight the imaging characteristics, underlying histopathology, and proposed clinical management, with the goal of improving understanding of this rare entity.

## 1. Introduction

Breast amyloidoma is an extremely rare condition initially described in 1973 [1] with few reported cases in the literature since then. Breast amyloidoma is characterized by localized deposition and accumulation of insoluble extracellular amyloid protein [2]. Interestingly, breast amyloidomas may arise as a focal manifestation of primary systemic amyloidosis or locally limited disease within the breast from regional deposition, with an equal prevalence between the two [3–6]. The clinical manifestations of breast amyloidomas are numerous, with a varied repertoire of presentations including fat necrosis mimics, painless solitary masses, lymphadenopathy, and breast cancer mimics [1–10]. Additionally, no consensus guidelines on standardized management of breast amyloidomas exist, owing to its clinical rarity. In this study, a case of isolated breast amyloidoma is presented for radiologic–pathologic correlation with the purpose of showcasing the radiographic and histopathologic findings of a condition that is suggested to be an underdiagnosed and occasionally misdiagnosed entity with indeterminate clinical implications [2, 6].

## 2. Case Presentation

**Figure 1:**
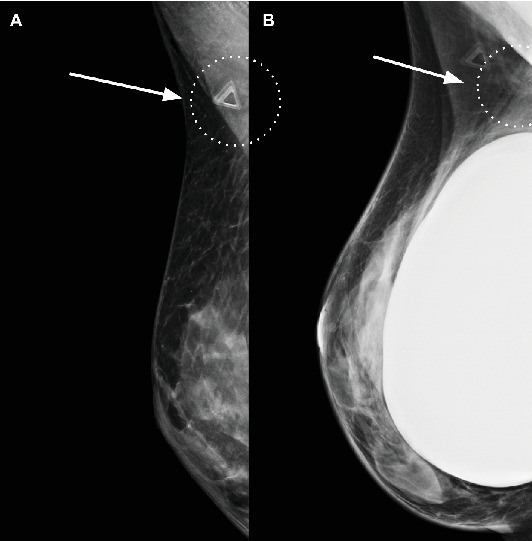
(A) Right mediolateral oblique implant-displaced and (B) right mediolateral oblique views of the right breast demonstrate an intact retroglandular silicone implant with background heterogeneously dense parenchyma. A triangular skin marker overlies the area of palpable concern. There is an irregular asymmetry with stippled calcification within the right axilla.

**Figure 2:**
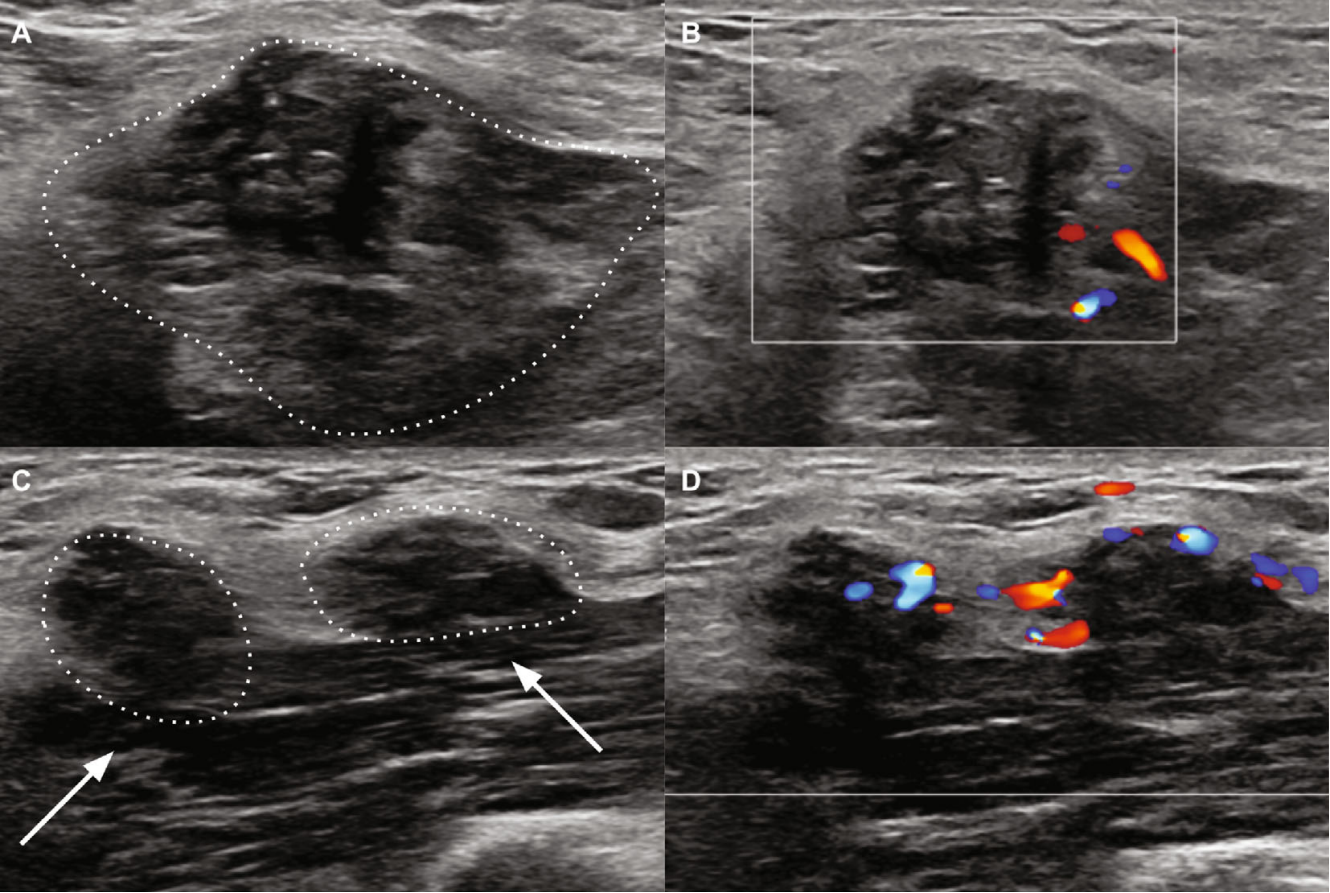
(A) Grayscale and (B) color Doppler images of the area of palpable concern within the right axilla demonstrate an irregular mass with indistinct margins and hypoechoic, heterogeneous internal echotexture. Calcifications are present in the mass, which overall measures 16 × 14 × 14 mm. (C) Grayscale and (D) color Doppler images of adjacent oval hypoechoic masses with indistinct margins, representing the same process.

A 46-year-old woman without significant past medical history presents to clinic for evaluation of a palpable right axillary breast mass. She denies any focal pain, nipple discharge, or retraction. Initial diagnostic imaging consisting of implant displaced and mediolateral oblique images of the right breast was obtained and demonstrated an irregular density with stippled calcifications within the right axilla (Figure 1). Further evaluation with dedicated sonographic imaging was performed, demonstrating an irregular hypoechoic axillary mass with indistinct margins and intrinsic calcifications, meeting the BI-RADS 4 criteria (Figure 2). The mass was subsequently interrogated via percutaneous core-needle biopsy sampling with results demonstrating breast amyloidoma with associated giant cell response. Congo red staining displayed amorphous fibrillary material with apple-green birefringence upon polarization. The patient was counseled regarding findings and pursued continued close clinical follow-up with their primary care provider.

## 3. Imaging Findings

The typical imaging characteristics of breast amyloidomas vary greatly and span the spectrum of benign fat necrosis mimics to breast cancer mimics [1, 3]. Breast amyloidomas commonly present as painless palpable masses and lack specific radiographic features [3–5]. They classically display a well-defined opacity with varying degrees of micro- or macrocalcifications, presumably due to focal accumulation of calcium-affixing properties of deposited amyloid protein in the perivascular and intralobular regions of the breast [2, 5, 7]. These masses may also demonstrate interval growth with increased calcifications as well as nonspecific axillary lymphadenopathy [2, 9], while other cases may not present with a mammographic correlate [11]. Sonographic characteristics also vary among reported cases and range from no sonographic correlate [7] to irregular hypoechoic or isoechoic avascular masses [5, 7, 11]. Overall, there is a lack of literature characterizing the sonographic appearance of breast amyloidomas. Magnetic resonance imaging characteristics of breast amyloidomas are also rarely reported in the literature, with two studies describing breast amyloidomas as nonenhancing masses with low-isointense signal on T1 and iso-hyperintense T2 signal [7, 8].

## 4. Pathologic Findings

Pathologic diagnosis of both local and systemic amyloidosis requires confirmation of amyloid deposits within tissues by direct sampling [11]. Additionally, diagnosis of systemic amyloidosis as systemic light chain type requires identification of free light chains in the serum or urine in addition to local tissue amyloid confirmation [11]. Congo red staining with demonstration of apple-green birefringence under polarized light remains the gold standard for identifying amyloid deposits with excellent sensitivity [11]. Histologically, amyloid may deposit within benign breast and lymphatic tissue but has also been reported in conjunction with mammary carcinomas and breast lymphoid malignancies [2]. In this presented case, histopathologic evaluation of the obtained tissue sample from the biopsied lesion demonstrated amorphous fibrillary material under Congo red staining, with apple-green birefringence upon polarization. Stains for lambda and kappa ISH, IgG, IgG4, IgA, and IgM were sent to assess background inflammatory cells, which demonstrated no focal abnormality. Additionally, serum protein electrophoresis and immunofixation demonstrated no monoclonal or polyclonal protein abnormality, and antinuclear antibody survey was also negative.

## 5. Discussion

Breast amyloidoma is an underdiagnosed and occasionally misdiagnosed entity due to its nonspecific radiographic appearance and occult histopathologic appearance requiring Congo red staining to establish the diagnosis [2, 6]. A certain level of suspicion may be necessary to prompt the specific staining needed for diagnosis. While breast amyloidomas are intrinsically benign lesions, their associations with systemic disorders, such as lymphomatous disease, systemic hematolymphoid malignancy, and systemic amyloidosis, demonstrate the need for more comprehensive management guidelines, particularly in individuals without these established diagnoses [2, 3]. Specifically, identification of breast amyloidomas in individuals without systemic disease should raise clinical suspicion for an underlying undiagnosed systemic disorder, given the preponderance of concurrent hematolymphoid malignancy of systemic amyloidosis in reported cases [2, 3]. This distinction is critically important due to the vastly different treatments for localized and systemic amyloidosis [2]. Localized disease may be managed with excision or expectant management with imaging follow-up. While a small retrospective review [2, 5] has suggested 6-month mammographic follow-up, the ideal time frame remains unclear due to indeterminate rates of recurrence [10]. Amyloidomas due to systemic disease, however, necessitate dedicated subspecialty consultation and portend a poorer prognosis than their local counterparts [5, 6]. Breast amyloidoma is suggested to be an underdiagnosed entity as evidenced by its nonspecific imaging and gross anatomic appearance, the few reported cases in the literature, and the lack of systematic utilization of Congo red staining during histopathologic evaluation of specimens [2, 6]. This is particularly relevant as accurate diagnosis and complete pathologic workup will ensure accurate diagnosis and prompt initiation of treatment while simultaneously avoiding unnecessary interventions for indeterminate cases [2]. Inclusion of breast amyloidomas in the differential diagnosis of suspected breast calcifications or masses and increased employment of Congo red staining is encouraged for this reason [2, 6]. In summary, we recommend the performance of larger-scale retrospective reviews and prospective cohort studies to further assess imaging features of breast amyloidomas, establish standardized management and follow-up guidelines, and recommend increased utilization of specific staining in suspected breast calcifications for improved diagnosis of this rare entity.

## Data Availability

The data that support the findings of this study are available on request from the corresponding author. The data are not publicly available due to privacy or ethical restrictions.

## Nomenclature

BI-RADS: Breast imaging-reporting and data system
